# Proactive use of eltrombopag before the onset of clinical bleeding in two children with immune thrombocytopenia and lifestyle restrictions

**DOI:** 10.1002/ccr3.1086

**Published:** 2017-09-01

**Authors:** Shayla Bergmann

**Affiliations:** ^1^ Department of Pediatric Hematology/Oncology Medical University of South Carolina 135 Rutledge Avenue MSC 558 Charleston 29425 South Carolina

**Keywords:** Hematology, pediatrics and adolescent medicine

## Abstract

Children with immune thrombocytopenia (ITP) are often managed using a watch‐and‐wait approach to avoid conventional treatment that may be poorly tolerated. However, in some patients, this approach may lead to lifestyle restrictions due to risk of injury‐related bleeding. Eltrombopag is a well‐tolerated new option that may help these children.

## Introduction

Immune thrombocytopenia (ITP) is the most common bleeding disorder in children 1. Most pediatric cases of ITP involve the immune system mistakenly attacking and destroying platelets. This process may be triggered by infection, primarily viral, certain medications, or other autoimmune conditions. Diagnosis of ITP is primarily based on the following: a history of isolated bleeding symptoms without constitutional symptoms; bleeding in the absence of hepatosplenomegaly, lymphadenopathy, or congenital conditions; isolated thrombocytopenia; and a peripheral smear with normal to large platelets and normal red and white blood cell morphology. Bone marrow evaluation is no longer necessary in patients with the typical features as ITP as stated. Patients often present with mild bleeding, including bruising, petechiae, and mucosal bleeding. However, bleeding can be fatal if it occurs in vital organs, but this rarely occurs (in 0.2% to 0.9% of patients) 2. ITP occurring for 3 months or less is termed acute or newly diagnosed, whereas persistent ITP is usually 3–12 months and it is chronic if it persists for more than 12 months 3. Children are more likely to have the acute form of ITP because thrombocytopenia spontaneously resolves within a year of diagnosis in most children (80%), regardless of therapy 4. However, in the remaining 20% of children, persistent thrombocytopenia despite therapy is discouraging and frustrating for the patient, family, and healthcare team.

A wide range of therapeutic regimens is currently in use for acute ITP, including intravenous gamma globulin (IVIG), steroids, and anti‐D immunoglobulin 5, 6. These treatments are usually reserved for patients with clinically significant bleeding as they are associated with serious adverse events 5, 6. If the patient's platelet counts (PCs) remain at a safe level (>20 × 10^9^/L [Gi/L]) with regard to hemostasis and in the absence of bleeding, observation without treatment is the typical management option 5, 6. However, it is usually recommended that children with PCs < 50 Gi/L avoid activities that may increase their risk of bleeding. This constraint may restrict their participation in sports and social and school activities, thereby impacting their lifestyles. Thus, children with chronic ITP may benefit from treatments that can maintain PC > 50 Gi/L. For many years, immunosuppressive treatments, including the standard therapies of acute ITP, were used in patients with chronic ITP. However, these treatments typically do not provide stable responses in the long term or are not well tolerated 5, 7, 8. Although splenectomy may be curative in the majority of patients with ITP, it is an irreversible surgical procedure and carries lifelong risks such as susceptibility to infections and thrombosis 2, 9.

Eltrombopag (Promacta^®^ – East Hanover, NJ in the United States; Revolade^®^ – Camberley, UK in the European Union) is a thrombopoietin receptor agonist that has been used to treat adult ITP for nearly a decade. Recently, the indication of eltrombopag in the United States and the European Union was expanded to include children ≥1 year of age with chronic ITP who have had an insufficient response to corticosteroids, immunoglobulins, or splenectomy 10, 11.

Here, we report our experience with eltrombopag in two pediatric patients with thrombocytopenia who did not benefit from standard treatments and had restrictions in their activities that significantly impacted their lives.

## Clinical Case 1

### Case history/examination

The first case is a very active 11‐year‐old boy participating in ice hockey and skateboarding who had a history of bipolar disorder and attention‐deficit/hyperactivity disorder (ADHD). In October 2015, he presented at a local urgent care center with a 3‐week history of easy bruising, fatigue, cold intolerance, and decreased appetite. He had a diffuse petechial rash that had started 1 day prior and was found to have a PC of 3 Gi/L. He was referred to our tertiary care center with a confirmed PC of 9 Gi/L. The remainder of his complete blood cell count (CBC) and his vital signs were normal.

### Differential diagnosis, investigations, and treatment

The patient had a previous CBC in May 2013 that revealed a PC of 84 Gi/L, but the family history was unremarkable for any hematologic or autoimmune disorders. He did not complain of any other symptoms, such as fevers, pain, shortness of breath or other respiratory symptoms, diarrhea, or vomiting. He had not been taking any nonsteroidal anti‐inflammatory drugs (NSAIDs) or antiplatelet agents, but he had been started on risperidone for bipolar disorder and guanfacine for ADHD about 11 weeks and 6 days prior to this presentation, respectively.

**Figure 1:**
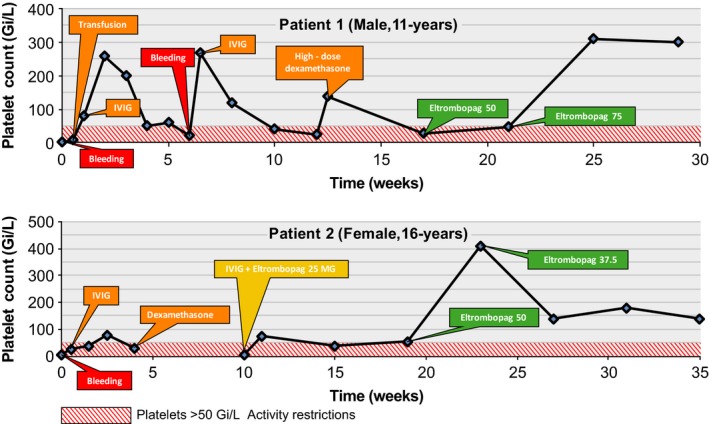
Platelet count over time. Time points of treatment initiation, subsequent treatments, and bleeding events are also shown. An appropriate dose of eltrombopag stabilized the platelet counts well above 50 Gi/L in both patients, eliminating activity restrictions and minimizing the risk of bleeding.

The patient was diagnosed with ITP possibly secondary to a drug reaction from risperidone or guanfacine. These medications were discontinued and treatment was started for ITP (please see Fig. 1A for the patient's PCs during the course of the treatment). After a failure to increase PCs above 7 Gi/L with a platelet transfusion, the patient received IVIG (1 g/kg), resulting in a PC of 80 Gi/L. The patient was subsequently discharged, and his PCs remained >50 Gi/L during the following 3 weeks. However, the patient had severe side effects from the IVIG infusions, including headache, fever, nausea, and vomiting.

One week after an outpatient follow‐up wherein his PC was 60 Gi/L, the patient returned with increased bruising and petechiae. The PC was 21 Gi/L, and the patient was treated with a second dose of IVIG (1 g/kg) that led to a PC increase to 268 Gi/L in 4 days. Severe side effects similar to the ones experienced during the first IVIG infusion recurred, and the PC fell back to 41 Gi/L within a month after infusion, leading to restriction of physical activities. At this point, anti‐Rho(D) immune globulin was offered as a treatment option, but the parents declined treatment to avoid hospital admission.

Two weeks after the second IVIG infusion, the patient returned for follow‐up. The PC was 24 Gi/L, and because the patient wished to go on a planned trip to a water park for his birthday, a third IVIG infusion was prescribed. However, vessel constriction possibly caused by mild dehydration and compounded by anticipation of multiple needle sticks prevented intravenous access, despite several attempts. Consequently, the parents agreed to a trial of high‐dose dexamethasone (40 mg/m^2^/day for 4 days). Although dexamethasone increased the PC to 137 Gi/L, it caused emotional lability and triggered some symptoms of his bipolar disorder.

Over the next month, his PC regressed to 20–30 Gi/L. He was again restricted from ice hockey, physical education at school, and any sort of risky physical activity. His depression was aggravated, and he started to have more frequent episodes of behavioral problems at home and school. Nevertheless, his parents wanted to avoid IVIG and steroids due to their severe side effects and wanted a well‐tolerated treatment option that would enable more stable responses. Accordingly, the patient was started on oral eltrombopag 50 mg once daily in mid‐February 2016.

### Outcome and follow‐up

The PC was improved from 26 to 47 Gi/L within 1 month. Because the patient wished to return to ice hockey full time and to have no activity restrictions, the dose of oral eltrombopag was increased to 75 mg once daily. His PC stabilized at approximately 300 Gi/L. The patient has been tolerating the medication very well without any side effects. He is playing hockey again. He is no longer mentally or emotionally labile. His daily life has greatly improved and is back to normal.

## Clinical Case 2

### Case history/examination

The second case is a 16‐year‐old girl with no significant medical history. She was an avid horseback rider and track runner and participated in a daily exercise program, and had a strong desire to maintain her active lifestyle. She presented in July 2015 after she noticed a large bruise on her left hip that was not caused by trauma as far as she remembered and small red spots on her legs. Her father, a physician, was concerned, and had her medically examined. Her PC was 5 Gi/L. She denied any shortness of breath or difficulty breathing, headaches, changes in vision or sensation, or pain. She had not had any other evidence of bleeding other than the bruises and petechiae.

### Differential diagnosis, investigations, and treatment

Her CBC (excluding PC), antineutrophil antibodies, rheumatoid factors, thyroid functions, immunoglobulins A, G, and M, and platelet antibodies were all normal, eliminating concern for malignant, rheumatologic, or immunologic confounding conditions. Of note, a paternal aunt had recurrent ITP about 2–3 years prior. She also has a cousin and a great‐uncle who died from acute myelogenous leukemia.

The patient had been using an oral contraceptive pill and daily doxycycline for her acne, but had stopped taking both about 2 weeks before admission. She had a normal menstrual cycle afterward without increased or prolonged bleeding.

The patient reported having had congestion, runny nose, and upset stomach with loose bowel movements her initial symptoms started 4–7 days before admission and she did not have any fevers or chills.

The patient was diagnosed with classic acute ITP following the viral illness as described. Indeed, pediatric cases of acute ITP commonly present a few weeks following infections, as infectious agents may stimulate production of antiplatelet antibodies resulting increased reticuloendothelial destruction of the platelets and thus thrombocytopenia. She received IVIG (1 g/kg), and her PC was 28 Gi/L 36 h after the dose (please see Fig. 1A for the patient's PCs during the course of the treatment). She suffered a severe headache for several days after the IVIG that required treatment with oxycodone.

Although her PC was 81 Gi/L 2 weeks after IVIG dosing, the count was back down to 29 Gi/L at her 1‐month follow‐up. Given her active lifestyle and strong interest in horseback riding and running track, we decided to proceed with further treatment and prescribed a 4‐day course of dexamethasone (40 mg once daily). However, 6 weeks later, her PC was 5 Gi/L. She then received IVIG (1 g/kg), and oral eltrombopag (25 mg once daily) was started.

### Outcome and follow‐up

After the IVIG infusion and initiation of eltrombopag, the patient's PC oscillated between 37 and 73 Gi/L over a 3‐month period (Fig. 1B). Considering the relatively high risk of injury associated with horseback riding, we adjusted our target platelet count to >100 Gi/L. The eltrombopag dose was increased to 50 mg once daily after 3 months of the first dose to stabilize the PC at >100 Gi/L.

A month following this dose increase, her PC rose to 410 Gi/L, and the dose was decreased to 37.5 mg once daily. She has been maintained for several months on this dose, with PCs between 140 and 182 Gi/L, and no side effects have been reported. She is riding horses daily and back to her regular exercise routine.

## Discussion

We described two children with ITP who had not had serious bleeding events but were experiencing severe lifestyle restrictions due to thrombocytopenia. The inability to participate in their desired sports resulted in emotional and mental stress, leading to aggravation of preexisting psychiatric problems in the first patient. Both patients have responded quite well to treatment with eltrombopag and are tolerating the medication without any side effects.

We recommend that CBCs and liver enzymes be regularly monitored in patients using eltrombopag, particularly during the dose adjustment phase. In one of our patients, platelet levels exceeded 410 Gi/L after a dose increase but resolved quickly after prompt detection with routine PCs and an appropriate dose adjustment. Although we did not observe any hepatic abnormalities in our patients, increased serum aminotransferases and bilirubin, which are typically reversible with dose adjustments, have been previously reported with eltrombopag 10.

These case reports demonstrate the meaningful use of eltrombopag in children with ITP who have experienced a perceivable negative impact on their lives due to the substantial restrictions in their activities but have not experienced serious bleeding events, an approach that has previously been suggested 12. Historically, for patients with no immediate risk of significant bleeding, an observe‐and‐wait approach was the only option in a setting where no well‐tolerated and effective long‐term treatments were available. However, we believe eltrombopag should be considered for this population as it may allow patients to return to their normal desired activities without restriction and eliminate the need for temporary treatments with substantial side effects.

## Conflict of Interests

Financial*:* Novartis Pharmaceutical Corporation, Speaker Program Participant. Baxalta Advisory Committee for The Treatment of Hemophilia, 2014. NonFinancial*:* None.

## Authorship

SB: collected, analyzed, and interpreted the clinical data, prepared the manuscript, and approved the final version to be published.

## References

[1] Terrell, D. R. , L. A. Beebe , S. K. Vesely , B. R. Neas , J. B. Segal , and J. N. George . 2010 The incidence of immune thrombocytopenic purpura in children and adults: a critical review of published reports. Am. J. Hematol. 85:174–180. 20131303 10.1002/ajh.21616

[2] Labarque, V. , and C. Van Geet . 2014 Clinical practice: immune thrombocytopenia in paediatrics. Eur. J. Pediatr. 173:163–172. 24390128 10.1007/s00431-013-2254-6

[3] Rodeghiero, F. , R. Stasi , T. Gernsheimer , M. Michel , D. Provan , D. M. Arnold , et al. 2009 Standardization of terminology, definitions and outcome criteria in immune thrombocytopenic purpura of adults and children: report from an international working group. Blood 113:2386–2393. 19005182 10.1182/blood-2008-07-162503

[4] Medeiros, D. , and G. R. Buchanan . 2000 Idiopathic thrombocytopenic purpura: beyond consensus. Curr. Opin. Pediatr. 12:4–9. 10676767 10.1097/00008480-200002000-00002

[5] Neunert, C. , W. Lim , M. Crowther , A. Cohen , L. Jr Solberg , and M. A. Crowther . 2011 The American Society of Hematology 2011 evidence‐based practice guideline for immune thrombocytopenia. Blood 117:4190–4207. 21325604 10.1182/blood-2010-08-302984

[6] Provan, D. , R. Stasi , A. C. Newland , V. S. Blanchette , P. Bolton‐Maggs , J. B. Bussel , et al. 2010 International consensus report on the investigation and management of primary immune thrombocytopenia. Blood 115:168–186. 19846889 10.1182/blood-2009-06-225565

[7] Arnold, D. M. 2013 Positioning new treatments in the management of immune thrombocytopenia. Pediatr. Blood Cancer 60(Suppl. 1):S19–S22. 23109488 10.1002/pbc.24341 PMC4854632

[8] Ghanima, W. , A. Khelif , A. Waage , M. Michel , G. E. Tjønnfjord , N. B. Romdhan , et al. 2015 Rituximab as second‐line treatment for adult immune thrombocytopenia (the RITP trial): a multicentre, randomised, double‐blind, placebo‐controlled trial. Lancet 385:1653–1661. 25662413 10.1016/S0140-6736(14)61495-1

[9] Kristinsson, S. Y. , G. Gridley , R. N. Hoover , D. Check , and O. Landgren . 2014 Long‐term risks after splenectomy among 8,149 cancer‐free American veterans: a cohort study with up to 27 years follow‐up. Haematologica 99:392–398. 24056815 10.3324/haematol.2013.092460 PMC3912973

[10] Novartis . 2015 Promacta [package insert]. Novartis Pharmaceuticals Corporation: East Hanover, NJ.

[11] Revolade (eltrombopag) . 2015 Summary of product characteristics. Novartis Europharm Limited: Camberley, UK.

[12] Cuker, A. , and C. E. Neunert . 2016 How I treat refractory immune thrombocytopenia. Blood 128:1547–1554. 27053529 10.1182/blood-2016-03-603365

